# SIRT1 Regulates Cognitive Performance and Ability of Learning and Memory in Diabetic and Nondiabetic Models

**DOI:** 10.1155/2017/7121827

**Published:** 2017-10-15

**Authors:** Yue Cao, Zi Yan, Tong Zhou, Guixia Wang

**Affiliations:** Department of Endocrinology and Metabolism, The First Hospital of Jilin University, Changchun, Jilin, China

## Abstract

Type 2 diabetes mellitus is a complex age-related metabolic disease. Cognitive dysfunction and learning and memory deficits are main characteristics of age-related metabolic diseases in the central nervous system. The underlying mechanisms contributing to cognitive decline are complex, especially cognitive dysfunction associated with type 2 diabetes mellitus. SIRT1, as one of the modulators in insulin resistance, is indispensable for learning and memory. In the present study, deacetylation, oxidative stress, mitochondrial dysfunction, inflammation, microRNA, and tau phosphorylation are considered in the context of mechanism and significance of SIRT1 in learning and memory in diabetic and nondiabetic murine models. In addition, future research directions in this field are discussed, including therapeutic potential of its activator, resveratrol, and application of other compounds in cognitive improvement. Our findings suggest that SIRT1 might be a potential therapeutic target for the treatment of cognitive impairment induced by type 2 diabetes mellitus.

## 1. Introduction

Type 2 diabetes mellitus (T2DM) is one of multiple age-related metabolic diseases [1]. Several latest studies have demonstrated severe and progressive abnormalities in brain structures and cognition during the early stage of T2DM [2]. T2DM is a risk factor for mild cognitive impairment (MCI) [3] and can accelerate the rate of functional decline in patients with mild dementia [4]. Cognitive dysfunction and learning and memory deficits have been considered one of the most prevalent and significant T2DM-related complications [4–8]. In recent years, silent information regulator 2 (Sir2), the highly conserved nicotinamide adenine dinucleotide- (NAD^+^-) dependent histone deacetylase [9], was shown to extend lifespan and delay aging in numerous studies ranging from *Saccharomyces cerevisiae* to mammals [10, 11]. As the ortholog of the yeast Sir2, SIRT1 is the most evolutionally conserved member [12]. Accumulating evidence has suggested that SIRT1 is expressed in the liver, skeletal muscle, pancreas, adipose tissues, and brain [13, 14], but its levels in the brain are notably higher than those in the other tissues in mammals [12, 15, 16], especially in the hippocampus, a vital structure closely related to learning and memory of the central nervous system [17]. SIRT1 participates in apoptosis [18], autophagy [19], and development [20], as well as in metabolism [21, 22] and circadian rhythms [23, 24]; therefore, it is not surprising that SIRT1 affects more complex biological processes including aging [24–27], MCI [28], and cognitive decline [29–31].

Present opinion on SIRT1 in cognition, learning, and memory is inconclusive. Some scholars believe that SIRT1 is positive for memory conservation. The spontaneous senescence-accelerated P8 mouse strain (SAMP8) is widely used as an animal model of aging [32–34] due to learning and memory deficits and behavioral alterations of Alzheimer's disease (AD) [35–39]. It has been demonstrated that the expression of SIRT1 declines with age in the brain of SAMP8 and senescence-accelerated mouse resistant 1 (SAMR1) [40], which have been extensively used as a control model because of the same genetic background and normal aging characteristics [41]. However, SIRT1 was decreased in the cerebral cortex and hippocampus [42] of SAMP8 mice [43, 44] compared with those of age-matched SAMR1. In addition, SIRT1 was downregulated in diverse models of cognitive impairment *in vivo* and *in vitro*, such as in juvenile C57BL/6J mice with dysmetabolism induced by high-caloric diet [45] and neurotoxic primary hippocampal neurons caused by toxins [44]. A study by Yokozawa et al. on antiaging effects of oligomeric proanthocyanidins found that SIRT1 was increased both in the cellular senescence model [46] and SAMP8 mouse model [47]. On the other hand, some researchers believed that SIRT1 has no effect on cognitive improvement and has a counterproductive effect. Most tellingly, earlier studies have proved that overexpression of SIRT1 may induce the memory deficit in transgenic (Tg) mice that overexpresses human SIRT1 in neurons [48]. Nicotinamide, an inhibitor of SIRT1, has been shown to attenuate cognitive deficits of 3xTg-AD mice via inhibition of SIRT1 and phosphorylation of tau [49]. Moreover, recent work has demonstrated that SIRT1 silencing could promote neuronal survival and protect neurons via the IGF-1 pathway [50].

Collectively, SIRT1 plays a significant role in learning and memory and provides enormous insights into T2DM-associated cognitive dysfunction. It is also rapidly emerging as a critical regulator of aging. However, positive or negative effects of SIRT1 on learning and memory have yet to be further discussed. In the ensuing paragraphs, we highlighted the involvement of SIRT1 in pathological processes of cognitive impairment in diabetic and nondiabetic models.

## 2. Role of SIRT1 on Cognition and Learning and Memory in Nondiabetic Models

### 2.1. Deacetylation of SIRT1

Many studies have confirmed that SIRT1 mediates chromatin silencing and chromatin remodeling through deacetylating histones, including H1, H3, and H4 [51] and modulates the activity of several protein targets that will be stated subsequently.

#### 2.1.1. SIRT1-p53 Pathway

It has been claimed that SIRT1 directly bound to and deacetylated p53 with specificity for its C-terminal Lys382 residue, inhibited acetylation of p53, and reduced the activity of downstream target genes [52, 53].

Decreased level of SIRT1 and increased level of acetylated p53 were observed in the hippocampal tissue [54] and cortex [55] of SAMP8 and *in vitro* studies [56]. Coincidentally, in juvenile C57BL/6J mice, low-caloric intake increased learning and memory function through positively downregulating p53 and unremarkably upregulating SIRT1 [45]. In the following studies, although no difference in SIRT1 level was detected between the control and the resveratrol dietary groups, researchers found that resveratrol improved learning and memory through the SIRT1-p53 pathway [57].

#### 2.1.2. SIRT1-AMPK Pathway

SIRT1 improves mitochondrial function by activating adenosine monophosphate-activated protein kinase (AMPK) through acetylating liver kinase B1 (LKB1) [58]. Conversely, AMPK improves SIRT1 activity by increasing cellular NAD^+^ levels to trigger the deacetylation of SIRT1 [59]. In the SAMP8 model, the increases in phosphorylated AMPK (p-AMPK) that regulate energy expenditure and the decreases in the production of reactive oxygen species (ROS) paralleled to the rise in SIRT1 in the hippocampus [54] and cortex [55]. Although without detection of LKB1, we suggest that this process might be triggered by SIRT1 deacetylation. In addition, in a rat model of AD with intracerebroventricular injection of streptozotocin (ICV-STZ) [60], the level of p-AMPK and SIRT1 activity were decreased and the level of phosphorylated tau was increased, while AMPK-specific activator prevented cognitive impairment through rescuing SIRT1 activity, downregulating tau hyperphosphorylation, and repairing mitochondrial function reflected by increased ATP levels, mitochondrial membrane potential, complex I activity, and SOD activity, as well as decreased ROS generation.

#### 2.1.3. Other Factors Deacetylated by SIRT1

In addition to the tumor suppressor factor p53 [61, 62] and serine-threonine protein kinase LKB1 [58, 63], SIRT1 deacetylated several transcriptional factors participated in transcriptional control of key genes in multiple cellular processes. These transcriptional factors regulate a wide range of metabolic activities, such as nuclear factor-kappa beta (NF*κβ*) [64], extracellular signal-regulated kinase (ERK) [65], the forkhead box subgroup O (FoxO) family [66, 67], peroxisome proliferator-activated receptors *γ* (PPAR*γ*), and its transcriptional coactivator PPAR*γ* coactivator 1-*α* (PGC-1*α*) [68, 69].

Direct *in vivo* evidence supported the link between SIRT1 and improvement of cognitive decline. The spatial memory deficit of ICV-STZ-treated rats was improved through ameliorating activation of SIRT1, which in turn attenuated tau phosphorylation by decreasing ERK1/2 phosphorylation [65]. In a study on neuroprotective role of intermittent fasting (IF) [70], upregulation of SIRT1 in the cortex and hippocampus of SAMP8 could possess neuroprotection via modulating downstream factors, including a decrease in phosphorylated Jun-terminal kinase (JNK), acetylated NF*κβ* [71], and acetylated FoxO1, as well as an increase in phosphorylated FoxO1. Additionally, in the hippocampus and cortex of SAMP8 mice [71] and in the hippocampus of 3xTg-AD mice [72], SIRT1 upregulated a disintegrin and metalloprotease 10 (ADAM10) [73] and downregulated the phosphorylated form of glycogen synthesis kinase 3 beta (GSK3*β*) [55, 70] in order to reduce the production of amyloid beta (A*β*) peptides and tau phosphorylation, which have been widely accepted as vital causes of cognitive decline [74]. SIRT1 was also noted to increase the expression of heat shock protein 70 (HSP70), a biomarker of neuronal survival, in SAMP8 models [70] and 3xTg-AD mice [72].

Moreover, an indirect proof of the effect of SIRT1 on cognition was demonstrated *in vitro*. In a study on neurite outgrowth and cell survival, SIRT1 was shown to promote neuronal growth through negative modulation of the mammalian target of rapamycin (mTOR)/p70S6 kinase (p70S6K) pathway in wild-type mouse primary neurons and human SIRT1 transgenic mice [75]. Furthermore, Codocedo et al. have suggested that SIRT1 accelerated the development and maintenance of dendritic branching in Sprague-Dawley rat primary hippocampal neurons by inhibiting the RhoA/Rho-associated protein kinase (ROCK) pathway and activating the Rac1/JNK pathway [76]. SIRT1was shown to attenuate glutamate-induced apoptosis in SH-SY5Y cells by upregulating PGC-1*α* [77].

**Figure 1:**
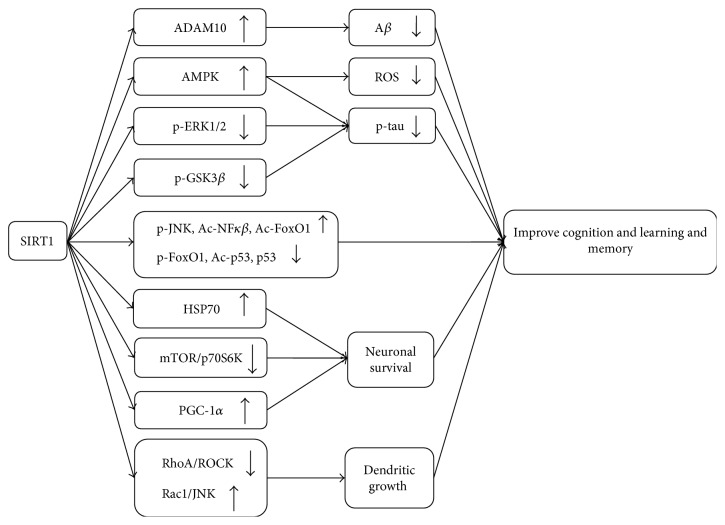
Deacetylation of SIRT1 in cognition and learning and memory. Increased SIRT1 level may reduce the production of ROS, A*β*, and p-tau, as well as promote neuronal survival and dendritic growth, contributing to improve learning and memory. ADAM10: a disintegrin and metalloprotease 10; AMPK: adenosine monophosphate-activated protein kinase; p-ERK: phosphorylated extracellular signal-regulated kinase; p-GSK3*β*: phosphorylated glycogen synthesis kinase 3 beta; p-JNK: phosphorylated Jun-terminal kinase; Ac-NF*κβ*: acetylated nuclear factor-kappa beta; Ac-FoxO1: acetylated the forkhead box subgroup O 1; Ac-p53: acetylated p53; HSP70: heat shock protein 70; mTOR: mammalian target of rapamycin; p70S6K: p70S6 kinase; PGC-1*α*: peroxisome proliferator-activated receptor *γ* transcriptional coactivator 1-*α*; ROCK: Rho-associated protein kinase; JNK: Jun N-terminal kinase; A*β*: amyloid beta; ROS: reactive oxygen species; p-tau: phosphorylated tau.

Such a point is worthy of further confirmation since growing evidence has indicated the presence of relationship between the role of SIRT1 on learning and memory and histone H2A variant, H2A.Z, which has been considered a negative regulator of consolidation of recent and remote memory [78]. H2A.Z was negatively regulated by the expression and activity of SIRT1 in some tissues [79] (Figure 1).

### 2.2. Targeting Oxidative Stress (OS)

As the basis of aging theories [80], OS can trigger the pathological processes of learning and memory deficits [81, 82]. A series of biomarkers represent the degree of OS, such as superoxide dismutase (SOD), reactive oxygen species (ROS), and malondialdehyde (MDA). Mitochondrial dysfunction is the central to oxidative damage and reflects the aging processes [83].

#### 2.2.1. Amelioration of Mitochondrial Dysfunction by SIRT1

There is a growing body of evidence supporting that mitochondrial dysfunction is critical for synaptic aging induced by chronic OS [82]. Data gathered from diverse studies have confirmed that oxidative stress could cause damages in the brain of SAMP8 mice [84, 85]. SAMP8 primary neurons had poor mitochondrial function, lower mitochondrial membrane potential, and higher mitochondrial vulnerability, all of which was protected by increased SIRT1 expression [56]. In addition, electron transport chain (ETC) related to mitochondrial oxidative phosphorylation (OXPHOS) was changed *in vitro* [56]. It was remarkable to find that SIRT1 could enhance OXPHOS via increasing the electronic chain-specific components ranging from complex I to complex V in the hippocampus of SAMP8 [54]. Consistent with this notion, SIRT1 improved spatial learning and memory deficits via SIRT1-mediated antioxidant signaling pathways in the D-galactose-induced aging rats [86]. Manganese superoxide dismutase (Mn-SOD) is an important antioxidative enzyme present in mitochondria. Recent data have shown downregulation of Mn-SOD mRNA levels by increasing level of SIRT1 [86].

#### 2.2.2. Interaction of ROS, Inflammatory Factor, and SIRT1

**Figure 2:**
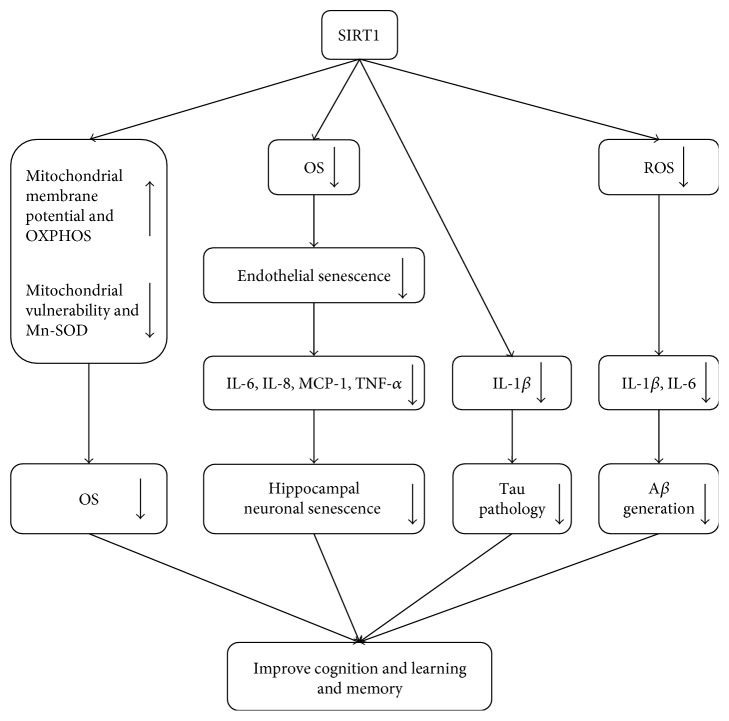
Oxidative stress (OS) is regulated by SIRT1 in cognition and learning and memory. SIRT1 may improve learning and memory by inhibiting OS, inhibiting the inflammatory response and hippocampal neuronal senescence, and decreasing the expression of tau and A*β*. OS: oxidative stress; OXPHOS: oxidative phosphorylation; Mn-SOD: manganese superoxide dismutase; IL-6: interleukin-6; IL-8: interleukin-8; MCP-1: monocyte chemoattractant protein-1; TNF-*α*: tumor necrosis factor-*α*; IL-1*β*: interleukin-1*β*; ROS: reactive oxygen species; A*β*: amyloid beta.

Lower levels of SOD, as well as higher levels of ROS [87], MDA [88], and some proinflammatory factors [87, 88], were found in SAMP8 compared with age-matched SAMR1. OS aggravated cognitive loss in SAMP8 models through either generating A*β*_1–40_ and A*β*_1–42_ by releasing interleukin-1*β* (IL-1*β*) and interleukin-6 (IL-6) [87] or enhancing neuroinflammatory activity by increasing IL-1*β*, tumor necrosis factor-*α* (TNF-*α*), and IL-6 [88]. In line with above evidence, *in vitro*, senescent endothelial cells induced by OS promoted the senescence of hippocampus neuronal cells through secretion of several inflammatory cytokines such as IL-6, interleukin-8 (IL-8), monocyte chemoattractant protein-1(MCP-1), and TNF-*α* [85]. Upregulation of SIRT1 could reverse inflammatory factors to rescue the production of A*β* and neuronal senescence [85, 87]. In addition, microglial SIRT1 deficiency elevated levels of IL-1*β* and exacerbated memory deficits in human P301S tau mice [29] exhibiting age-dependent synaptic loss and tau-mediated memory deficits [89]. All above have demonstrated that neuroinflammatory played a significant role in learning and memory modulated by SIRT1 (Figure 2).

### 2.3. SIRT1-microRNA Pathway

It is assumed that cyclic AMP response element-binding protein (CREB), a molecular switch of long-term memory that maintains cognitive function [90], binds to several promoters of brain-derived neurotrophic factor (BDNF) and regulates its expression. Recent studies have shown that SIRT1 promotes plasticity and memory in a direct manner via a miR-134-mediated posttranscriptional mechanism. The results suggested that SIRT1 cooperated with Yin Yang 1 (YY1) in binding to the upstream regulatory elements of miR-134 and then limited the expression of miR-134 resulting in overexpression of CREB and BDNF, thereby regulating synaptic plasticity and long-term memory formation in SIRT1-KO mice [91]. Additionally, resveratrol was shown to improve learning and memory in normally aged C57BL/6J mice through the SIRT1-microRNA pathway [92]. Furthermore, SIRT1 increased the expression of BDNF in SAMP8 models [70] and 3xTg-AD mice [72]. In the hippocampus of rats receiving lead exposure, SIRT1 and CREB phosphorylation were decreased in a dose-dependent manner, which could be reversed by resveratrol [93]. Resveratrol also ameliorated spatial learning memory impairment induced by A*β*_1–42_ in rat hippocampus by elevating SIRT1 expression and CREB phosphorylation [31]. Although miR-134 was not detected in the above three studies, we still suggested that SIRT1 protects learning and memory via the SIRT1-miR-134 pathway.

## 3. The Mechanism of SIRT1 on Cognition and Learning and Memory under the Condition of Insulin Resistance (IR)

In the above section, we have summarized the role of SIRT1 in cognitive dysfunction and learning and memory deficits under normal physiological condition. Next, we will discuss its role under the condition of insulin resistance. It is widely known that caloric restriction (CR) has benefits on cognition decline [94]. Emerging evidence has indicated a causal link between T2DM and cognition decline and learning and memory deficits [3–5, 95–97], such as MCI [98]. The mechanisms that trigger learning and memory deficits in diabetic models include inflammation [99], loss of neuronal plasticity [100, 101], alteration of mitochondrial structure and function [102, 103], elevation of cerebral A*β*, and tau phosphorylation [100]. Therefore, cognitive ability is distinctly affected by metabolic status.

**Figure 3:**
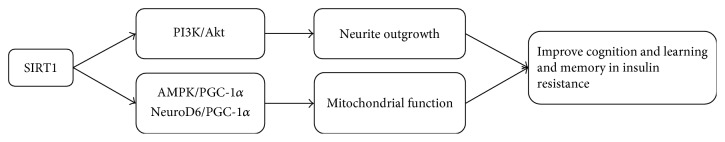
The role of SIRT1 in cognition and learning and memory under the condition of IR. SIRT1 has been shown to increase neurite outgrowth by activating the PI3K/Akt pathway and improve mitochondrial function through the AMPK/PGC-1*α* and NeuroD6/PGC-1*α* pathways. PI3K: phosphoinositide 3-kinase; AMPK: adenosine monophosphate-activated protein kinase; PGC-1*α*: PPAR*γ* coactivator 1-*α*; NeuroD6: neuronal differentiation 6.

Accumulating evidence has indicated the inhibition of SIRT1 protein expression and activity in T2DM or IR [104–107]. Data has shown that activated SIRT1 improves the insulin sensitivity of the liver, skeletal muscle, and adipose tissues, as well as protects the function and cell mass of pancreatic *β*-cells [13]. So, does SIRT1 involve in it? And whether SIRT1 regulates learning and memory directly or indirectly? Next, we set forth the role of SIRT1 in cognition and learning and memory under the condition of IR (Figure 3).

### 3.1. SIRT1 Promotes Neurite Outgrowth

Several studies have demonstrated that SIRT1 modulates neuronal viability [36, 37], neuronal differentiation [38–41], neuronal protection [42–44], and synaptic plasticity [21, 45–47], all of which are key factors largely linked to cognitive improvement. It is well established that insulin exerts its actions in a series of biological processes through binding to insulin receptors [108], as well as plays an essential role in IR and T2DM. Recently, researchers have demonstrated that insulin-induced neurite outgrowth is regulated by SIRT1, which is dependent on the PI3K/Akt signaling pathway in SH-SY5Y cells [109]. In accordance with the above views, we suggest that SIRT1 may be imbalanced when insulin signaling is impaired and cause an influence on cognition and neurodegeneration.

### 3.2. SIRT1 Improves Mitochondrial Function in the Brain

SIRT1 activation has a significant coordinating role in mitochondrial function. It is noteworthy that NeuroD6, as a regulator of ROS homeostasis [110], is related to learning and memory. As a marker of mitochondrial biogenesis, PGC-1*α* may take part in cognitive decline under metabolic stress. Moreover, AMPK is a sensor key that controls PGC-1*α* activity. In the aged C57BL/6J mouse model of IR induced by a high-fat diet, SIRT1 improved mitochondrial function via the SIRT1-AMPK-PGC-1*α* axis and the neuronal differentiation 6 (NeuroD6)-PGC-1*α*-SIRT1 axis to enhance cognitive decline [102]. Coincidentally, the SIRT1-AMPK-PGC-1*α* pathway was also verified in the SAMP8 model of IR induced by a high-fat diet, albeit the levels of SIRT1 were not significantly modified [103].

A study by Lennox et al. has demonstrated that increased SIRT1 enhanced cognitive function and synaptic plasticity via alleviating IR in high-fat-fed mice [111]. Similarly, upregulated SIRT1 simultaneously improved synaptic plasticity and insulin signaling in the hippocampus and cortex of high-fat-fed mice [112]. Although few studies have examined the association between IR and cognitive impairment, we concluded that SIRT1 might contribute indirectly to improve cognition, because many of SIRT1's downstream regulators are involved in memory processes.

## 4. The Effects of Resveratrol on Cognition and Learning and Memory

### 4.1. Resveratrol, Targeting SIRT1 or Not?

Resveratrol has attracted considerable attention for its effects on the improvement of IR [113], cognitive decline [31, 65, 114–116], and cardiovascular diseases [117, 118]. Although resveratrol is widely accepted as a natural activator of SIRT1, there is also evidence showing that resveratrol may not be the direct agonist of SIRT1.

*In vitro*, resveratrol regulates brain function through increasing the biogenesis of *α*-amino-3-hydroxy-5-methyl-4-isoxazolepropionic acid receptor (AMPAR), a glutamatergic receptor, mediated by AMPK and subsequent downstream PI3K/Akt signaling in rat primary neurons [119]. *In vivo*, resveratrol improves learning and memory through activating the IGF-1-PI3K-p-CREB signaling pathway in the hippocampal CA1 region of juvenile and healthy C57BL/6J mice [57], while maintaining the same expression level of SIRT1. In the same strain with isoflurane-induced cognitive impairment, Li et al. have found that resveratrol exerts anti-inflammatory and antiapoptotic actions to recover cognition without alteration of SIRT1 [120]. In their work, the factors related to neuroapoptosis were changed, such as downregulation of cleaved caspase-3 and Bax and upregulation of Bcl-2. Meanwhile, NLRP3, an intracellular receptor of inflammatory responses, IL-1*β*, and TNF-*α* were decreased.

The effects of resveratrol on cognitive improvement are likely not to be fully dependent on SIRT1. After treatment with resveratrol, activation of the Wnt/*β*-catenin pathway by increasing GSK-3*β* might as well protect cognitive disturbances in diabetic C57BL/6J mice [102] and SAMP8 mice under the condition of metabolic stress induced by a high-fat diet [103]. In the latter study, Palomera-Avalos et al. have put forward that resveratrol improves mitochondrial morphology, dynamics, and OXPHOS via a decrease in mitofusin 2 (MFN2) and an increase in optic atrophy-1 protein (OPA1), I-NDUFB8, II-SDNB, III-UQCRC2, V-ATPase complexes, and voltage-dependent anion channel 1 (VDAC1)/porin [103].

### 4.2. Resveratrol, Improving Cognition and Learning and Memory or Not?

According to above notions, resveratrol plays a significant role in cognitive enhancement. However, its effects on cognition and learning and memory are still controversial.

#### 4.2.1. The Effects of Resveratrol on Animal Models

In several studies, the administration of oligomeric proanthocyanidins [47], pterostilbene [121], and rapamycin [122] displayed antiaging effects, whereas resveratrol did not show a marked effect. In the aspect of improvement of spatial learning and memory, five-week resveratrol administration to SAMP8 mice (90 *μ*mol/kg body weight/day, about equal to 20 mg/kg body weight/day) showed no significant changes compared to oligomer administration with the same period (50 mg/kg body weight/day). Similarly, eight-week pterostilbene administration to SAMP8 mice (120 mg/kg body weight/day) exerted beneficial effects on learning and memory, but not resveratrol at an identical dose for 8 weeks. In the aspect of survival, resveratrol administration (50 mg/kg body weight/day and 200 mg/kg body weight/day) did not extend life span of genetically heterogeneous mice, while low-dose rapamycin-treated mice (2.24 mg/kg body weight/day) showed an increase in the life span.

#### 4.2.2. The Effects of Resveratrol in Clinical Trials

Data of several clinical trials about resveratrol acting on cognition and learning and memory has indicated that resveratrol plays a protective role not only in diabetic patients but also in nondiabetic population except for patients with schizophrenia. In a randomized controlled trial on T2DM adults, a low dose of resveratrol (75 mg at weekly intervals) showed a positive but chronic effect on cerebrovascular function and cognitive function [123, 124]. In a 14-week randomized placebo-controlled intervention trial, resveratrol supplementation (75 mg twice daily) improved cognitive performance, mood, and cerebrovascular function in postmenopausal women [125]. However, resveratrol supplementation (200 mg/day for 1 month) did not improve memory and attention in 19 men with a diagnosis of schizophrenia [126].

Taken together, their findings have indicated that dosage and period of treatment may influence the effects of resveratrol. Compared to previous studies on antiaging and protection of cognitive decline, dosage of resveratrol and period of treatment are both insufficient. This idea suggests that resveratrol may not be the optimal choice for improvement of learning and memory in short-term treatment. So far, preclinical and clinical data in this area are limited, and an in-depth study of resveratrol on learning and memory needs to be further investigated.

## 5. Conclusion

SIRT1 may improve cognition and learning and memory through several pathways including deacetylation, OS, mitochondrial dysfunction, and inflammation and microRNA. However, the mechanisms of SIRT1 in cognitive decline under the condition of IR are inadequate and not in-depth and systematical, for example, a gap in the studies on mechanism of SIRT1 regulating neuronal energy metabolism and function. Meanwhile, the involvement of resveratrol, an activator of SIRT1, in the protection of cognitive deficits is still not completely clear. But in most cases, resveratrol can improve cognition and learning and memory. Thereby, the neuroprotection of SIRT1 and resveratrol and their interaction should be explored to act on preventing cognitive impairment in T2DM-associated cognitive dysfunction. In brief, SIRT1 may provide potential approaches to improve learning and memory. Long-term therapy of large-dose resveratrol may offer therapeutic possibilities for preventive strategies in T2DM-associated cognitive dysfunction.
